# Pancreatic Secretory Trypsin Inhibitor (SPINK1) Gene Mutation in Patients with Acute Alcohol Pancreatitis (AAP) Compared to Healthy Controls and Heavy Alcohol Users without Pancreatitis

**DOI:** 10.3390/ijms232415726

**Published:** 2022-12-11

**Authors:** Anssi Nikkola, Kari Antero Mäkelä, Karl-Heinz Herzig, Shivaprakash Jagalur Mutt, Aishwarya Prasannan, Hanna Seppänen, Terho Lehtimäki, Mika Kähönen, Olli Raitakari, Ilkka Seppälä, Pihla Pakkanen, Isto Nordback, Juhani Sand, Johanna Laukkarinen

**Affiliations:** 1 Department of Gastroenterology and Alimentary Tract Surgery, Tampere University Hospital, 33520 Tampere, Finland; anssi.nikkola@fimnet.fi (A.N.); juhani.sand@pshp.fi (J.S.); 2 Faculty of Medicine and Health Technology, Tampere University, 33014 Tampere, Finland; terho.lehtimaki@tuni.fi (T.L.); mika.kahonen@tuni.fi (M.K.); pihla.pakkanen@hus.fi (P.P.); isto.nordback@pshp.fi (I.N.); 3 Research Unit of Biomedicine, Oulu University, 90220 Oulu, Finland; kari.makela@oulu.fi (K.A.M.); karl-heinz.herzig@oulu.fi (K.-H.H.); shivaprakash.jagalurmutt@mcb.uu.se (S.J.M.); aishwarya.prasannan@oulu.fi (A.P.); 4 Medical Research Center Oulu, Oulu University, Oulu University Hospital, 90220 Oulu, Finland; 5 Department of Pediatric Gastroenterology and Metabolic Diseases, Poznan University of Medical Sciences, 60-572 Poznan, Poland; 6 Department of Surgery, Helsinki University Hospital, 00260 Helsinki, Finland; hanna.seppanen@hus.fi; 7 Fimlab Laboratories, Department of Clinical Chemistry, 33520 Tampere, Finland; ilkka.seppala@tuni.fi; 8 Finnish Cardiovascular Research Center, 33520 Tampere, Finland; 9 Department of Clinical Physiology, Tampere University Hospital, 33520 Tampere, Finland; 10 Centre for Population Health Research, University of Turku and Turku University Hospital, 20521 Turku, Finland; olli.raitakari@utu.fi; 11 Research Centre of Applied and Preventive Cardiovascular Medicine, University of Turku, 20014 Turku, Finland; 12 Department of Clinical Physiology and Nuclear Medicine, Turku University Hospital, 20521 Turku, Finland; 13 Department of Otorhinolaryngology-Head and Neck Surgery, Helsinki University Hospital, 00260 Helsinki, Finland

**Keywords:** acute alcohol pancreatitis, alcoholics, genetics, genes, SPINK1, serine protease inhibitor Kazal-type 1

## Abstract

Only 3–5% of heavy alcohol users develop acute alcohol pancreatitis (AAP). This suggests that additional triggers are required to initiate the inflammatory process. Genetic susceptibility contributes to the development of AAP, and SPINK1 mutation is a documented risk factor. We investigated the prevalence of the SPINK1(N34S) mutation in patients with AAP compared to heavy alcohol users who had never suffered an episode of pancreatitis. Blood samples for the mutational analysis from patients with first episode (*n* = 60) and recurrent AAP (*n* = 43) and from heavy alcohol users without a history of AAP (*n* = 98) as well as from a control population (*n* = 1914) were obtained. SPINK1 mutation was found in 8.7% of the patients with AAP. The prevalence was significantly lower in healthy controls (3.4%, OR 2.72; 1.32–5.64) and very low in alcoholics without pancreatitis (1.0%, OR 9.29; 1.15–74.74). In a comparison adjusted for potential cofounders between AAP patients and alcoholics, SPINK1 was found to be an independent marker for AAP. The prevalence of the SPINK1 mutation is overrepresented in AAP patients and very low in alcoholics without pancreatitis. This finding may play a role in understanding the variable susceptibility to AAP found in heavy alcohol users.

## 1. Introduction

Acute pancreatitis (AP) is a common gastrointestinal disease with an average incidence of 30–50/100,000 inhabitants in western countries [1,2]. Gallstones are considered the most common etiology for AP with alcohol being the second [3,4]. Currently, alcohol is the number one etiology in Finland with around 70% of cases being alcohol induced [5]. Heavy alcohol consumption is one of the most important risk factors for first episode and recurrent AP (RAP). Epidiemiologic evidence shows that the risk of AP correlates with increasing alcohol consumption and binge drinking [6,7]. Amount, type and duration of alcohol consumption to initiate the inflammatory process is still unclear [8]. It has been demonstrated that abstinence effectively protects against recurrences in acute alcohol pancreatitis (AAP) [9,10]. However, only around 3%–5% of heavy alcohol users develop AAP regardless of heavy drinking [11,12]. This suggests that there are additional triggers that are required to initiate the inflammatory process in AP. It may also be that lack of protective factors increases the susceptibility to AP. 

Regarding other risk factors for AAP, smoking has been shown to elevate the risk for first and recurrent non-gallstone-related AP and longer duration of smoking seems to be associated with an even more increased risk [13,14,15,16]. Obesity has been demonstrated to increase the risk for both non-gallstone- and gallstone-related AP [17,18,19,20]. Type 2 diabetes mellitus (T2DM) increases the risk for AP and simultaneous alcoholism potentiates the risk [20]. Genetic predisposition such as X-chromosome-linked claudin-2 (CLDN2) mutation as well as drinking habits may explain why AAP is more common in men [21]. The first attack of AAP typically presents in the age between 30–60 years [22].

In AP, the premature activation of trypsinogen to trypsin and the consequent pancreatic autodigestion results in a local inflammatory process. Genetic susceptibility to pancreatitis was first discovered in 1996 with the cationic trypsinogen (PRSS1) mutation in patients with hereditary pancreatitis (HP) [23]. Since then, numerous genetic variants have been linked to pancreatitis. One of the most studied is the mutation in serine protease inhibitor Kazal-type 1 (SPINK1). SPINK1 is an antiprotease which serves as an inactivator of prematurely activated trypsin and therefore protects against pancreatitis. The association between SPINK1 mutation and chronic pancreatitis (CP) was first discovered in 2000, and subsequently shown in AP and hereditary pancreatitis [24,25,26]. According to a recent meta-analysis, SPINK1 mutation significantly increases the risk of AP [27]. SPINK1 may also be associated with more severe AP, but this finding remains uncertain [25,28].

Although several genes have been documented as risk factors for pancreatitis, only a few genetic studies have been conducted in the context of environmental and clinical factors. Our aim was to study the prevalence of SPINK1 (N34S) mutation in relation to known risk factors for non-gallstone pancreatitis in patients with AAP compared to heavy alcohol users who had never suffered an episode of pancreatitis.

## 2. Results

Baseline characteristics are shown in Table 1. The mean alcohol consumption (19.5 vs. 10.3 portions per sitting, *p* < 0.001) and Alcohol Use Disorders Identification Test (AUDIT) scores (30.9 vs. 18.5, *p* < 0.001) were significantly higher in the alcoholics group compared to AAP patients. Alcoholics were significantly more frequently smokers compared to AAP patients (82.7% vs. 60.2%, *p* = 0.001) and healthy controls (82.7% vs. 14.5%, *p* < 0.001). Alcoholics used drugs more often compared to AAP patients (10.2% vs. 4.9%, *p* = 0.03). 

**Figure 1:**
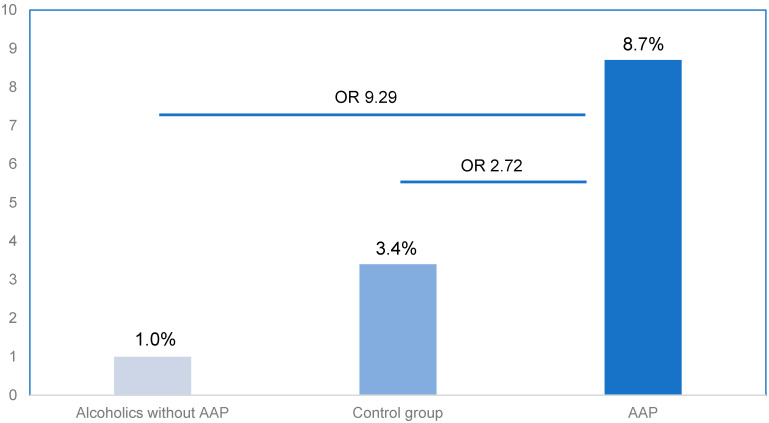
The prevalence and odds rations of SPINK1 (N34S) in patient and control groups.

The SPINK1 N34S mutation was found in 8.7% (*n* = 9) of the patients with AAP. The prevalence of SPINK1 did not significantly differ between first and recurrent AAP (10% vs. 7.0% respectively, *p* = 0.435). Of the 103 AAP patients, the disease was mild in 77 (74.8%), moderately severe in 22 (21.4%) and severe in 4 (3.9%) cases. In all patients with SPINK1 mutation, the severity of AP was mild. However, compared to patients with a normal variant, the difference in severity did not reach statistical significance (p = 0.084). As shown in Figure 1, the prevalence of SPINK1 mutation was significantly lower in healthy controls (3.4%, *n* = 65: OR 2.72; 1.32–5.64) and very low in alcoholics without pancreatitis (1.0%, *n* = 1: 9.29; 1.15–74.74). The difference remained statistically significant when comparing only patients with first episode AAP to the control population (OR 3.16; 1.31–7.61), as well as to the alcoholics group (OR 10.78; 1.26–91.89).

In a binomial logistic analysis comparing AAP patients with the control population adjusted for smoking, alcohol consumption, BMI, gender and T2DM (Table 2), the SPINK1 mutation did not remain as an independent marker in AAP (OR 2.36; 0.40–14.02). In this model, alcohol consumption (daily intake g/day: OR 2.11; 1.85–2.40), male sex (OR 3.08; 1.29–7.39) and smoking (OR 2.60; 1.29–5.22) were found to be independent markers in AAP. Other cofounders were not statistically significant. When using the same model comparing AAP patients and heavy alcohol users, SPINK1 was found to be an independent marker in AAP (OR 26.32; 2.52–274.41) and male sex (OR 12.96; 4.54–36.96). In this comparison model, amount of alcohol consumed was found to have a negative correlation (OR 0.83; 0.78–0.88).

## 3. Discussion

To the best of our knowledge, this is the first study comparing the prevalence of SPINK1 N34S mutation in AAP patients, alcoholics without history of pancreatitis and healthy controls in the context of known risk factors for AAP. Our report suggests that in AAP patients SPINK1 mutations are prevalent in almost 9% of cases, which is significantly more than in healthy controls or alcoholics without pancreatitis. In contrast to our expectations, SPINK1 mutations were not an independent risk factor for AAP. However, in a subgroup of individuals with excessive alcohol use, it was found to be independently associated with AAP.

Mutation in SPINK1 N34S is an accepted risk factor for pancreatitis [25,27]. Testing for SPINK1 gene mutations is recommended in AP patients with recurrent idiopathic pancreatitis, patients under 25 years with RAP, and patients with a positive family history [29]. In patients with high burden of risk factors, genetic testing including SPINK1 mutation analysis may be useful to elucidate further risk factors. After an episode of AAP, interventions against alcoholism and the associated risk factors have been shown to prevent recurrent attacks [30]. 

Our study’s strengths include amount and preciseness of data on known risk factors for AAP in patients and alcoholics groups. We accounted for the main risk factors for AAP and demonstrated that in individuals with excessive drinking, the burden of known risk factor is not directly associated with the development to AAP. We demonstrate that in alcoholics without pancreatitis, drinking habits (daily intake in grams per day, portions per sitting, AUDIT points and type of alcohol consumed) were significantly higher compared to AAP patients. They were more active smokers and used more drugs than AAP patients. Our data suggest that these individuals who do not develop AAP despite the heavy burden of risk factors have less other risk factors and/or protective factors against the disease. It has been proposed that alcohol, as well as smoking, serves as a disease modifier and that accumulation of risk factors, including increased genetic susceptibility, is needed to initiate the inflammatory cascade in AP [31]. 

In our study, BMI of alcoholics was significantly lower than in patients with AAP (25.4 vs. 26.9). This difference did not remain significant in the multivariate analysis. Additionally, most of the AAP patients were males. Drinking patterns and amount of alcohol consumed in males do at least partly explain the sex difference [11]. However, other susceptibility factors, such as X-chromosome-linked CLDN2 mutation has been proposed as a possible factor [21]. In a comparison between AAP patients and alcoholics without pancreatitis, we demonstrate that the role of SPINK1 mutation is overrepresented as an individual risk factor for AAP. As only a minority of heavy alcohol users develop AAP, our findings demonstrate that genetic predisposition plays a significant role in determining the risk of AAP. However, it seems that SPINK1 only partially explains the variable susceptibility to AAP found in heavy alcohol users. It would be interesting to study the role of other known genetic risk alleles in a genome-wide association study. The lack of wider genetic analysis is a limitation of our study as well as our relatively small study population.

In our large control population, SPINK1 N34S mutation was found in 3.4% of the individuals, which is consistent with previous reports from Finland [25,32]. The associating between SPINK1 mutation and pancreatitis has been well established. In a meta-analysis of 42 studies by Liu et al., the pooled OR for pancreatitis in total was 7.77 (95% CI, 5.23–11.54) [33]. Regarding the associating between AP and SPINK1 N34S, a meta-analysis by Jøergensen et al. yielded an OR of 2.82 (2.03–3.93) [34]. Aoun et al. found that SPINK1 was not associated with the initial attack of AP [35]. In patients with AAP, we demonstrated a significant association between SPINK1 mutation and the first episode of AAP. Abstinence has been shown to effectively protect against recurrences of AAP [9,10]. Since the etiology of AP was exclusively alcohol-induced in our study, it is probable that some patients after the initial attack of pancreatitis develop recurrent attacks later if abstinence is not achieved, especially if they have SPINK1 mutation.

## 4. Materials and Methods

In a prospective study, the study population consisted of 103 patients with AAP, of whom 60 had their first and 43 had a recurrent episode. The patients were treated at Tampere University Hospital which is the only tertiary care hospital in Pirkanmaa Hospital District, Finland. The diagnosis of AP was based on at least two of the following three criteria: (1) Serum amylase level more than three times the upper reference limit, (2) Acute onset epigastric pain, (3) Suitable findings in imaging studies (computed tomography, ultrasonography, magnetic resonance imaging) according to the 2012 revised Atlanta Classification [36]. Alcohol as a probable etiology of pancreatitis was determined by self-reported history of heavy drinking, suitable laboratory testing (carbohydrate-deficient transferrin, mean corpuscular volume, and glutamyltransferase) and using the Alcohol Use Disorders Identification Test (AUDIT). Type of alcohol consumed was also recorded and divided into mild (≤22% of ethyl alcohol by volume, ABV) and hard alcohol (>22% respectively).

EDTA blood samples from patients with AAP were obtained during hospitalization or during subsequent control visit and stored at –70 °C. Hospital records, including laboratory testing results, imaging findings and medical records including alcohol use (AUDIT testing, average alcohol intake in grams per day in the last 4 weeks and reported portions per sitting; amount of alcoholic portions containing an equivalent of 14 g of pure alcohol on an average day in the last 4 weeks), as well as illicit drug (any illicit drug use, self-reported) and tobacco use (currently smoking or in the last 3 years, total years of smoking, cigarettes per day) were collected. From a local A-clinic (alcohol and substance abuse prevention and treatment clinic), we collected the same baseline information including substance usage (alcohol, smoking, illicit drugs) and obtained EDTA blood samples from the individuals during their A-clinic visits (alcoholics group, *n* = 98). Data from patients with AAP were collected between January 2004 and November 2012 and from alcoholics between March 2004 and November 2005. A Finnish control population from five University Hospital districts (Turku, Helsinki, Tampere, Kuopio and Oulu areas) of 1914 individuals was previously collected as a part of a genome-wide association study (GWAS, Young Finns study) [37]. Their genome was previously analyzed.

### 4.1. DNA Extraction and Purification from Blood

Genomic DNA was extracted from 200 µL of whole blood using MACHEREY-NAGEL NucleoSpin^®^ Blood kit (Düren, Germany) according to manufacturer´s instructions. The samples were eluted in 60 µL of elution buffer. DNA was stored in −70 °C for further analysis. 

### 4.2. Detection of Spink1 Mutation

Primer sequences for detection of N34S mutation in the SPINK1 gene (NCBI Reference Sequence NG_008356.2) were adapted as described previously [38]. The sequences were the following: forward primer 5′-CTC TTA CTG GAG TAG AAT GCA-3′, reverse primer 5′-GTT TGC TTT TCT CGG GGT GAG-3′. PCR reactions contained 2.5 µL of 10X DreamTaq Buffer (includes 20 mM MgCl_2_; ThermoFisher Scientific, Waltham, MA, USA), 0.5 µL of dNTP mix (10 mM each; ThermoFisher Scientific), 1 µL of each primer (10 µM), 2 µL of template and 0.125 µL of DreamTaq DNA Polymerase (5 U/µL; ThermoFisher Scientific) with sterile water that was used to adjust the final volume to 25 µL. The following cycling conditions were used: 95 °C for 3 min (initial denaturation), 95 °C for 30 s, 54 °C for 30 s, 72 °C for 1 min (cycling repeated 36 times) and final extension at 72 °C for 2 min. A total of 20 µL of PCR product was digested with 3 µL of HpyCH4III restriction enzyme (5 U/µL; New England Biolabs, Ipswich, UK) by following manufacturer´s instructions. Digested samples were run in commercial 5% polyacrylamide Mini-PROTEAN TBE Precast Gels (Bio-Rad Laboratories, Hercules, CA, USA). The N34S mutation produces two additional bands at 109 and 189 bp by cutting the 298 bp fragment present in wild-type samples among other fragments [38].

### 4.3. Statistical Analysis

Statistical analysis was carried out using IBM SPSS Statistics for Macintosh and Windows (Version 26.0, Armonk, NY, USA). The *p*-values under 0.05 were considered statistically significant. Fisher’s exact test and Chi-square test were used in categorical variables when appropriate and student’s *t*-test (normal distribution) or Mann–Whitney U test (skewed distribution) were used in continuous variables to test for statistical significance. Odds ratios were calculated using 95% confidence interval. Effect of SPINK1 mutation as an independent factor for AAP was evaluated using binomial logistic regression model adjusted for potential confounders.

## 5. Conclusions

SPINK1 N34S mutation is overexpressed in AAP patients compared to healthy controls and the prevalence was remarkably low in alcoholics without pancreatitis. In patients with excessive alcohol use, the expression of SPINK1 mutation seems to be an independent risk factor for AAP. These findings may help in assessing the individual risk for pancreatitis as well as to effectively target interventions against recurrent attacks.

## Figures and Tables

**Table 1:** Demographic and clinical characteristics of alcoholics without pancreatitis, AAP patients and control population.

	Alcoholics	*p*-Value	AAP	*p*-Value	Controls
	(*n* = 98)		(*n* = 103)		(*n* = 1914)
Mean age (years)	54 (30–76)	0.761	54 (25–88)	<0.001	42 (34–49)
Sex; female (%)	31/98 (31.6)	<0.001	9/103 (8.7)	<0.001	1067/1914 (55.7)
Mean BMI	25.4 (16–40) a	0.014	26.9 (18–46)	0.416	26.5 (16–58) b
T2DM (%)	2 (2)	0.098	7 (6.8)	0.009	37 (2) c
Smoking (%)	81/98 (82.7)	0.001	62/103 (60.2)	<0.001	264/1817 (14.5) d
Years of smoking	23.4 (0–45)	0.042	19.4 (0–52)	NA	NA
Cigarettes per day	19 (0–60)	0.001	12 (0–90)	NA	NA
Illicit drug use (%)	14/98 (10.2)	0.03	5/103 (4.9)	NA	NA
Alcoholic portions per sitting	19.5 (5–52)	<0.001	10.3 (1–30)	NA	NA
Alcohol intake g/day	149.7 (2.6–624.0)	<0.001	60.7 (1.7–312.0) e	<0.001	9.0 (0.0–171.4) f
Type of Alcohol					
Mild, ABV ≤ 22% (%)	27 (27.5)	0.001	58 (56.3)	NA	NA
Hard, ABV > 22% (%)	22 (22.4)	0.986	23 (22.5)	NA	NA
Mixed (%)	49 (50)	<0.001	22 (21.4)	NA	NA
Mean AUDIT	30.9 (15–40) g	<0.001	18.5 (1–40) h	NA	NA

a: *n* = 95, b: *n* = 1903, c: *n* = 1772, d: *n* = 1817, e: *n* = 97, f: *n* = 1877, g: *n* = 91, h: *n* = 101.

**Table 2:** Known risk factors for AAP in a binomial regression analysis comparing AAP patients with alcoholics and healthy controls.

Variable	OR (CI 95%)	OR (CI 95%)
	Alcoholics vs. AAP (*n* = 98 & 103)	Controls vs. AAP (*n* = 1914 & 103)
Smoking	2.06 (0.86–4.91)	2.60 (1.29–5.22)
Alcohol intake g/day	0.83 (0.78–0.88)	2.11 (1.85–2.40)
BMI	1.08 (0.99–1.19)	1.01 (0.94–1.09) ^a^
Gender (male)	12.96 (4.54–36.96)	3.08 (1.29–7.39)
T2DM	6.92 (0.54–88.84)	1.92 (0.39–9.43)
SPINK1 mutation	26.32 (2.52–274.41)	2.36 (0.40–14.02)

a: *n* = 95, BMI not recorded in 8 AAP patients.
